# Ampholytic and Polyelectrolytic Starch as Matrices for Controlled Drug Delivery

**DOI:** 10.3390/pharmaceutics11060253

**Published:** 2019-06-01

**Authors:** Nassim Benyerbah, Pompilia Ispas-Szabo, Khalil Sakeer, Daniel Chapdelaine, Mircea Alexandru Mateescu

**Affiliations:** Department of Chemistry, Research Chair in Enteric Dysfunctions “Allerdys”, CERMO-FC Center, Université du Québec à Montréal, C.P. 8888, Branch A, Montréal, QB H3C 3P8, Canada; benyerbah.nassim@courrier.uqam.ca (N.B.); ispas-szabo.pompilia@uqam.ca (P.I.-S.); sakeer.khaleel@courrier.uqam.ca (K.S.); chapdelaine.daniel@uqam.ca (D.C.)

**Keywords:** ampholytic starch, polyelectrolytic starch, electrostatic stabilization, high drug loading, drug controlled release, spray drying

## Abstract

The potential of the polyampholytic and polyelectrolytic starch compounds as excipients for drug controlled release was investigated using various tracers differing in terms of solubility and permeability. Ampholytic trimethylaminecarboxymethylstarch (TMACMS) simultaneously carrying trimethylaminehydroxypropyl (TMA) cationic groups and carboxymethyl (CM) anionic groups was obtained in one-step synthesis in aqueous media. Trimethylaminestarch (TMAS) and carboxymethylstarch (CMS) powders were also synthesized separately and then homogenized at equal proportions in liquid phase for co-processing by spray drying (SD) to obtain polyelectrolytic complexes TMAS-CMS (SD). Similarly, equal amounts of TMAS and CMS powders were dry mixed (DM) to obtain TMAS:CMS (DM). Monolithic tablets were obtained by direct compression of excipient/API mixes with 60% or 80% drug loads. The in vitro dissolution tests showed that ampholytic (TMACMS) and co-processed TMAS-CMS (SD) with selected tracers (one from each class of Biopharmaceutical Classification System (BCS)), were able to control the release even at very high loading (80%). The presence of opposite charges located at adequate distances may impact the polymeric chain organisation, their self-assembling, and implicitly the control of drug release. In conclusion, irrespective of preparation procedure, ampholytic and polyelectrolytic starch materials exhibited similar behaviours. Electrostatic interactions generated polymeric matrices conferring good mechanical features of tablets even at high drug loading.

## 1. Introduction

Oral solid dosages (i.e., tablets, capsules) have always been among the most studied galenic forms in pharmaceutical development. The interest in modifying their release led to sustained investigation efforts aimed to develop new technologies, formulas, or devices to control the drug release. The expectations and, in a large measure, the achievements of drug delivery systems (DDS) were to optimize the treatments by administering precise doses (avoiding under/overdose) [1], targeting the affected areas and improving the patient compliance [2,3]. Orally controlled release systems (OCRS) can be obtained by: osmotic delivery systems [4,5], porous structured materials [6], tablet coating [7,8], and controlled release from monolithic matrices (using functional excipients) [9]. Such excipients may be synthetic (i.e., plycarbonates, polyesters, or polyamides) [10], or based on natural products (i.e., chitosan, cellulose, or starch) able to modify the release time of active ingredients in the digestive tract [11].

Starch is one of the most studied biopolymers known for its biocompatibility, biodegradability, and abundant sources as well as for the different possibilities of modification (i.e., substitution, reticulation) offered by its hydroxyl groups (–OH) [12]. Starch and its derivatives are largely used as excipients for dry galenic formulation [10]. Starch was often modified in order to modulate its physicochemical properties. Crosslinked starch was commercialized either as matrix for controlled release (Contramid^®^) of active pharmaceutical ingredient (API) [13] and, at different crosslinking degrees, as binder and disintegrant (Liamid^®^) [14]. Despite their similar backbone, their different degrees of crosslinking influence the organization of chains leading to different behavior in the drug release [15].

Starch excipients functionalized by substitution of -OH as starch acetate (SAc), aminoethyl starch (AES) or carboxymethyl starch (CMS) excipients have different features depending on their chemical nature [3,16]. The CMS obtained by etherification of high amylose starch with sodium monochloroacetate has been extensively studied for biomedical applications [17,18,19,20,21]. Its self-assembling by non-covalent inter and intra-molecular ionic and hydrogen interactions between the carbohydrates chains led to new structural conformations and more complex organization [9]. CMS has a pH-dependent character that offers protection by protonation/deprotonation phenomena [3]. This behavior allowed the use of CMS as a matrix for bioactive agents [3] such as enzymes [18,22], microorganisms [16], and probiotics for intestinal delivery [23].

Generally, the starch derivatives can be synthesized either in organic or inorganic media. The drying step can be carried out by precipitation (methanol/acetone), lyophilization, or spray drying. A correlation between the DS, the drying method, and the physicochemical properties of the CMS was previously shown [24]. The CMS was co-processed with other polymers such as chitosan [25] to generate polyelectrolyte complexes (PEC). Differently to PECs, ampholytic starch derivatives are simultaneously carrying two types of functional groups of opposite charges on the carbohydrate chains [26]. There are a few repported applications of such derivatives as flocculating agents for water treatment [27,28] or in the textile and paper industries [27]. Ampholytic starch was described with quaternary ammonium and phosphate groups [27], carboxymethyl groups [29], or succinate [30]. Another starch material with aminoethyl and carboxymethyl groups (CMAESt) was apparently the first ampholytic starch used as an excipient in controlled drug release systems [26,31]. The results for monolithic tablets with 60% API loading showed that the impact of ionic interactions improved the stabilization of polysaccharide chains.

In the present study, a new form of ampholytic starch was investigated. This new derivative carries quaternary amine and carboxymethyl groups. It was synthesized by simultaneous starch treatment with glycidyltrimethylammonium chloride and sodium chloroacetate to obtain trimethylaminecarboxymethylstarch (TMACMS). Separately, starch derivatives carrying only cationic groups: trimethylaminestarch (TMAS) or anionic groups: carboxymethylstarch (CMS) were also synthesized. For a better understanding of mechanisms governing the drug release from these new types of starch derivatives, the impact of the physical processing on the release of selected active molecules was also explored. Thus, the anionic and the cationic starch derivatives were mixed by two different methods to obtain polyelectrolytes: 1) co-processing by spray drying (SD) of the aqueous mixtures of TMAS and CMS to obtain polyelectrolyte complexes abbreviated as TMA-CMS (SD) or 2) by dry mixture (DM) of powders of each compound at room temperature, abbreviated as TMA-CMS (DM). All derivatives were structurally characterized by Fourier transform infrared spectroscopy (FTIR), X-ray diffraction (DRX), thermogravimetric analysis (TGA), scanning electron microscopy (SEM), as well as by micromeritic studies. All new products were used as excipients for monolithic tablets with model active molecules having different solubilities and ionic charges.

## 2. Materials and Methods

### 2.1. Materials

High amylose starch (Hylon VII) was supplied by National Starch/ Ingredion (Westchester, IL, USA). Mesalamine (pharmaceutical grade) was obtained from PharmaZell (Raubling, Germany). Naproxen and atenolol were purchased from Sigma-Aldrich (Oakville, ON, Canada). Sodium monochloroacetate (SMCA) was from Sigma-Aldrich (Taufkirchen, Germany). Glycidyltrimethylammonium chloride (GTMAC) and acetaminophen were from Sigma-Aldrich (St. Louis, MO, USA).

### 2.2. Methods

#### 2.2.1. Synthesis of Starch Derivatives

The aim of synthesis was to obtain three excipients based on modified starch: two polyelectrolyte starch derivatives carrying carboxylic groups –COO^−^ (CMS) with negative overall charges or carrying quaternary amine groups –N^+^(CH_3_)_3_ (TMAS) with positive overall charges. The polyelectrolytic complexes were produced by i) co-processing by spray drying (SD), ii) homogenization of powders by direct physical mixing (dry mixed (DM)), iii) the third product was: TMACMS, an ampholytic starch carrying carboxylic groups (CM) and quaternary amine groups (TMA) on the same starch backbone synthesized in an one-step chemical process by adding simultaneously SMCA and GTMAC to the gelatinized starch. To synthesize polyelectrolytic complexes, CMS and TMAS were prepared separately using almost the same steps as in previous described by Sakeer et al. [32], slightly modified as follows: 25 g of native starch (Hylon VII) were dispersed in 200 mL of distilled water under stirring and 300 mL of 5 M NaOH were added continuing the stirring for 1h to obtain a gelatinized starch. Then an amount of 18.75 g of SMCA, rapidly dissolved in a minimal water volume was added to prepare CMS. In the same conditions, a volume of 24.4 mL of GTMAC was added to the gelatinized starch to obtain TMAS. The reaction was continued to be stirred at 60 °C for 1h. In both cases the solution was cooled down rapidly to 4 °C and the pH was decreased to 7 with glacial acetic acid. Each modified starch was precipitated with a solution of methanol:distilled water (70:30 *v*/*v*) and washed by decantation with the same solution until the preparation had a conductivity below 100 μS/cm. The slurry was then washed by filtration with pure acetone for final drying under vacuum. The obtained CMS and TMAS powders were left overnight at room temperature and then sieved to retain particles smaller 300 µm. Ampholytic starch TMACMS was prepared by the chemical process as previously described by Sakeer et al. [31]. Briefly, same amounts of SMCA and GTMAC were simultaneously added to gelatinized starch under continuous stirring at 60–70 °C for 1h. The solution was cooled down and pH was decreased to 7 with glacial acetic acid. Modified starch was precipitated with a solution of methanol:distilled water (70:30 *v*/*v*) and washed by decantation with the same solution until the preparation had a conductivity lower than 100 μS/cm. The slurry was then washed by filtration with pure acetone for final drying under vacuum. The powder was left overnight at room temperature and only particles smaller than 300 µm were retained after sieving. The polyelectrolyte TMAS-CMS (SD) was obtained separately: 10 g of CMS were completely dissolved in 1 L distilled water (solution 1). Separately, 10 g of TMAS were completely dissolved in 1L distilled water (solution 2). Solutions 1 and 2 were mixed and spray drayed (Buchi mini spray dryer B-290, Flawil, Switzerland) at 200 °C. The obtained TMAS-CMS (SD) powder was left overnight in an oven at 40 °C to eliminate all humidity. The polyelectrolyte blend TMAS:CMS (DM) was obtained by direct mixing of powders (50:50 *w*/*w*) for homogenization.

#### 2.2.2. Degree of Substitution (DS)

The degree of substitution at level of –OH by carboxymethyl groups (DS_coo-_) of CMS was determined by back titration. An amount of 200 mg of polymer was solubilized in 20 mL of NaOH (0.05 M) and the excess of NaOH (unreacted with carboxylic groups) was titrated by HCl (0.05 M). Color change of the indicator phenolphthalein was determined as the equivalence point. Twenty milliliters of NaOH (0.05 M) were also titrated and used as blank. All the titrations were done in triplicate. The DS_coo-_ was obtained by calculating the amount of carboxylic groups as follows:

$$X=\left(V_{b}-V_{s}\right)\times C_{HCl}$$

$$DS_{coo-}=\frac{162X}{m-58X}$$

where *V_b_* and *V_s_* are the volumes (L) of HCl (0.05 M) for titration of blank and samples respectively; *C_HCl_* is the concentration of HCl (mol/L); 162 (g/mol) molecular weight of one glucose unit; 58 (g/mol) is the increase in the weight of one glucose unit after substitution of hydroxyl groups by carboxymethyl group, and *m* (g): mass of dry powders. The degree of substitution of hydroxyl group by trimethyl amine group (DS **_N_**^+^_(CH3)3_) of TMAS and TMACMS were calculated by elemental analysis. Results were given by percentage of molecular mass for each element.

#### 2.2.3. Fourier Transform Infrared (FT-IR)

FTIR spectra of Hylon VII, CMS, TMAS, and polyelectrolytic starch powders were recorded by a Thermo Scientific, Nicolet 6700/Smart iTR (ThermoFisher Scientific, Madison, WI, USA) using a diamond crystal with 64 scans/min at 4 cm^−1^ resolution in the spectral region (4000–500 cm^−1^).

#### 2.2.4. X-ray Diffraction

The polymorphism of powders was evaluated by X-ray diffractometry using a Bruker, D8 Advance (Munich, Germany) device. A copper cathode (Cu Kα) was used in reflectance mode with a scanning rate of 0.05°/min. All patterns were analyzed through a 2θ range of 5°–50°. EVA software was used for X-ray diffraction spectra treatments.

#### 2.2.5. Zeta Potential (ζ)

The zeta potential of polymers was measured by ZetaPlus/Bl-PALS (Brookhaven Instrument Corp, Holtsville, NY, USA). Polymers were dissolved (0.01% *w*/*v*) in nonopure water. The analysis of particle surface charges was carried out at 25 °C using 1 mL of suspension. Measurements were done in triplicates.

#### 2.2.6. pH Determination

A Cole-Parmer PC200S pH/mV/Cond (Cole-Parmer, Chicago, IL, USA) was used to measure the pH of sample suspension (polymer: nanopure water 2.5% *w*/*v*) at room temperature. All measurements were done in triplicates.

#### 2.2.7. Scanning Electron Microscopy (SEM)

The morphology of powders of native starch (Hylon VII) and of starch derivatives was investigated using a Hitachi (S-4300SE/N) scanning electron microscopy with variable pressure (Hitachi High Technologies America, Pleasanton, CA, USA). Powder samples were put on a support covered by a double-sided carbon tape and sputter-coated with gold. The magnification was 100× and 1000× for all samples using.

#### 2.2.8. Thermogravimetric Analyses (TGA)

The weight loss depending on the variation of temperature was measured by a Q500 TGA Thermogravimeter (TA Instruments, New Castle, DE, USA) between 30 and 700 °C at 10 °C/min in a nitrogen atmosphere at 100 mL/min, using a small amount of powder filed on a platinum crucible. The analysis was carried out until the complete disappearance of the samples. “TRIOS” software was used for treatment of results.

#### 2.2.9. Micromeritic Analyses

Micrometric tests are used to study physicochemical properties of the obtained powders; they have been carried out according to USP recommendations using a Tap Density Tester (Vankel, Varian, NC, USA). The powder’s characteristics were studied using <1174> USP method [33] and the angle of repose (*θ*) was determined using the fixed funnel method [34] and calculated with the equation:

$$\theta =tan^{-1}\left(\frac{h}{r}\right)$$

where *h* stands for height; and *r* for ray.

The powder was poured into a funnel fixed 2 cm from a flat surface. The passage of the powder formed a cone. “*r*” is the ray of the base and “*h*” is the height of the cone (2 cm). Compressibility index (CI) and Hausner’s factor (HF) values were obtained according to the ˂616˃ USP [33] by measuring tapped density (ρ_tab_) and bulk density (ρ_bulk_). Briefly, a known mass of powder is placed in a graduated cylinder, the corresponding volume (mL) is noted. The ratio mass (g) volume (mL) is called bulk density which represents the density of a powder including the spaces between the particles. Tapped density was obtained by tapping the same cylinder using Tap Density Tester (Vankel, Varian, NC, USA) until there was a new volume of powder (elimination of space between the particles). Tapped density was represented by ratio mass (g):volume (mL). CI and HF were calculated as follows:

$$CI=\frac{\rho_{\mathrm{tab}}-\rho_{bulk}}{\rho_{tab}}\times 100$$

$$HF=\frac{\rho_{tab}}{\rho_{bulk}}$$

#### 2.2.10. Preparation of Tablets

To determine swelling, fluid uptake, and erosion, drug-free tablets (placebos) were prepared. CMS, TMAS, and polyelectrolytic or ampholytic starch powders were directly compressed (2.5 T/cm^2^) using flat-faced punches with 12.9 mm diameter (Carver hydraulic press, C 3912 Hydraulic Cylinder. Wabash, IN, USA) to obtain monolithic round tablets. For in vitro dissolution assays, the same excipients were used to prepare 12.9 mm diameter monolithic tablets with two different loadings. For acetaminophen or naproxen, 500 mg of API powders were homogenized with 333 mg or 125 mg of powdered polymers to obtain respectively 60% and 80% API loaded tablets. For mesalamine, 400 mg of API were homogenously mixed with 267 mg or 100 mg of polymers to obtain respectively 60% and 80% API loaded tablets. For atenolol, 9 mm flat-faced punch was used because of the small amount of powder: 200 mg of API powders were homogenized with 133 mg or 50 mg of powdered polymers to obtain respectively 60% and 80% atenolol loaded tablets. For all APIs, equal amounts of TMAS and CMS powders were dry mixed (DM) to obtain TMAS:CMS (DM).

#### 2.2.11. Determination of Swelling, Fluid Uptake and Erosion

Drug-free tablets have been used to investigate the behavior of starch compounds in simulated biological fluids: simulated gastric fluid (SGF) and simulated intestinal fluid (SIF). First, dry tablets were weighed (*W*_1_) and their diameter and thickness were measured. Then they were immersed in 40 mL of SGF (pH 1.2) and submitted to rotation at 50 rpm (Glas-Col rotator, Terre Haute, IN, USA) for 2 h. The tablets were then transferred in 40 mL SIF (pH 6.8) and submitted to rotation at 50 rpm. After every 2 h, tablets were withdrawn and weighed at time t (*W*_t_), diameters and thickness were also measured. Finally, tablets were placed in an oven at 40 °C for 48 h to obtain completely dry tablets (W_f_).

$$\text{Fluid uptake equation}:\;\%\;Weight\;change=\frac{W_{t}-W_{1}}{W_{1}}X100$$

$$\text{Degree of erosion equation}:\;\%\;Erosion=\frac{W_{1}-W_{f}}{W_{1}}X100$$

where *W*_1_ is the weight of the dry tablets; *W*_t_ the weight of the tablets at time *t*; and *W*_f_ the final weight of the tablets.

#### 2.2.12. In Vitro Dissolution Tests

In vitro drug release tests have been done following USP requirements for all active agents using a Distek dissolution system 2100A (Markham, ON, Canada) as USP paddle apparatus II. Acetaminophen tablets (60% and 80% loading) were immersed in 900 mL SGF at 50 rpm for 2 h and then transferred in 900 mL SIF at 50 rpm. At a predetermined sampling interval (1 h), volumes of 3 mL were withdrawn and filtered before spectrophotometric reading at λ_243nm_. Mesalamine tablets (60% and 80% loading) were immersed in 500 mL SGF at 100 rpm for 2 h and then transferred in 900 mL SIF at 50 rpm. At a predetermined sampling interval, volumes of 3 mL were withdrawn and filtered before a spectrophotometric reading in SGF at λ_300nm_ and in SIF at λ_330nm_. Naproxen and atenolol tablets (60% and 80% loading) were immersed in 900 mL SGF at 50 rpm for 2 h and then transferred in 900 mL SIF at 50 rpm. At a predetermined sampling interval, volumes of 3 mL were withdrawn and filtered before spectrophotometric reading in SGF at λ_224nm_ for naproxen and λ_225nm_ for atenolol, and in SIF at λ_232_ nm for naproxen and λ_225nm_ for atenolol. For each analysis, withdrawn volumes were immediately replaced. After each sampling, an equivalent volume (3mL) of dissolution medium was added to the dissolution cell. Standard curves of each drug were used to determine the concentrations of released drugs.

## 3. Results

### 3.1. Structural Properties

The degree of substitution (DS), which represents the average number of hydroxyl groups substituted with anionic functional groups (DS_coo-_) or with cationic groups (DS_N_^+^_(CH3)3_) per glucose unit, was determined by back titration and elemental analysis. The DS_coo-_ was 0.113 for CMS and 0.097 for TMACMS. The DS_N_^+^_(CH3)3_ was 0.195 for TMAS and 0.160 for TMACMS. Solutions of 2.5% in distilled water were prepared for the measurement of pH, the results obtained were: 6.70 for CMS, 7.01 for TMAS, 7.24 for TMACMS, 6.76 for TMAS-CMS (SD), and 7.11 for TMAS:CMS (DM). The zeta potential (ζ) for CMS was ζ = −36.44 mV and for TMAS was ζ = +30.20 mV. The bifunctional starch TMACMS had ζ = +17.26 mV. The zeta potential of TMAS-CMS (SD) and of TMAS:CMS (DM) was -26.28 mV and −32.42 mV respectively.

### 3.2. Fourier Transform Infrared (FT-IR)

**Figure 1:**
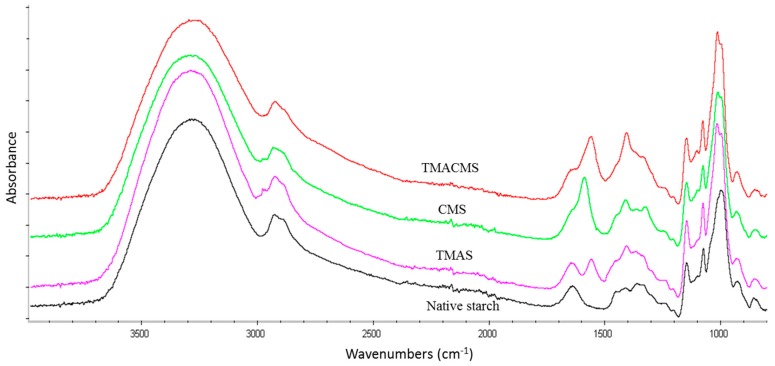
FTIR spectra of native starch (black), trimethylaminestarch (TMAS) (pink), carboxymethylstarch (CMS) (green), and of trimethylaminecarboxymethylstarch (TMACMS) (red).

The FTIR of CMS is characterized by two specific bands at 1589 and 1415 cm^−1^ (Figure 1). For TMAS, a new band at 1553 cm^−1^ and a shoulder at 1476 cm^−1^ are detected compared with FTIR spectrum of Hylon VII. Bands at 3331 cm^−1^, 2980 cm^−1^, 1089 cm^−1^, and at 1000 cm^−1^ were common for native starch and all derivatives. The FTIR spectrum of ampholytic starch TMACMS shows bands at 1553 cm^−1^ and at 1415 cm^−1^ (Figure 1).

### 3.3. X-ray Diffraction Analysis

**Figure 2:**
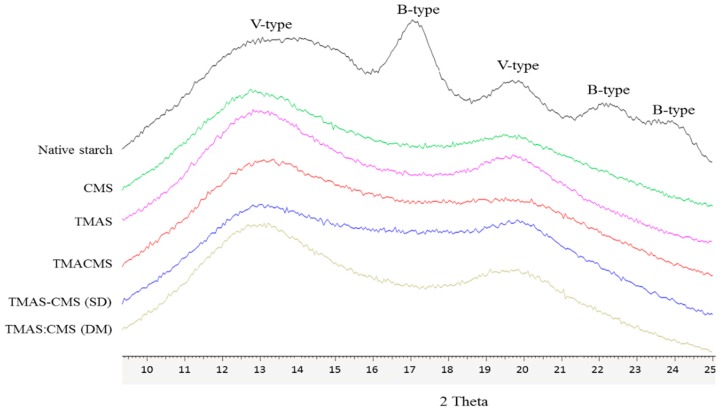
X-ray diffraction patterns of native starch (Hylon VII), and of CMS, TMAS, TMACMS, TMAS-CMS (spray drying (SD)), and TMAS:CMS (dry mixed (DM)) starch products.

The X-ray diffraction carried out on powders of Hylon VII and of obtained starch compounds are shown on Figure 2. Hylon VII presents peaks at 17.09°, 22.13°, 23.52°, 24.08°, 12.9°, and at 19.89°. The spectra of TMAS, CMS, TMACMS derivatives and of compounds TMAS-CMS (SD) and TMAS:CMS (DM) showed only two broader major peaks at 12.91° and 19.89°.

### 3.4. Thermogravimetric Analysis (TGA)

**Figure 3:**
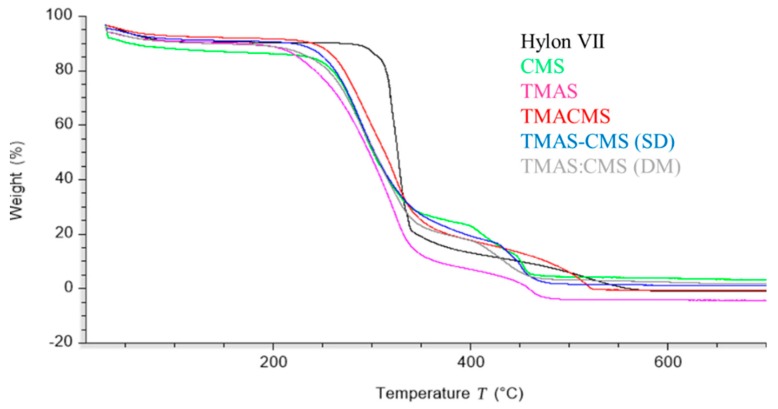
Thermogravimetric patterns of Hylon VII, CMS, TMAS, TMACMS, TMAS-CMS (SD), and TMAS:CMS (DM).

The thermal decomposition of native starch and of its derivatives was studied by thermogravimetric analysis (Figure 3) in the range of 30–700 °C. A first limited loss of mass between 60–120 °C was observed for all samples. The second sharp decomposition was found below 350 °C followed by the last stage between 350–500 °C. Native starch exhibited the highest decomposition temperature (TD) 327 °C while CMS (287 °C), TMACMS (291 °C), TMAS-CMS (SD) (287 °C), and TMAS:CMS (DM) (292 °C) display neighboring TD.

### 3.5. Scanning Electron Microscopy (SEM)

**Figure 4:**
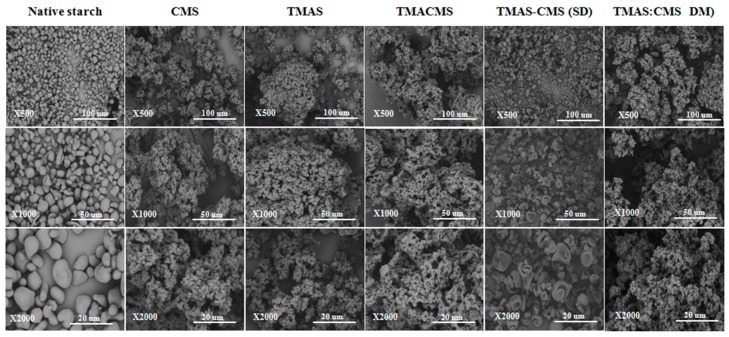
Scanning electron microscopy images of native starch, CMS, TMAS, TMACMS, TMAS-CMS (SD), and TMAS:CMS (DM) at magnifications ×500, ×1000, and ×2000.

Morphologies of native starch and of compounds were investigated by scanning electron microscopy (SEM) at three magnifications and some significant differences were observed (Figure 4). The native starch is formed by well distinct smooth granules of different sizes in irregular round or oval shapes. Differently, CMS, TMAS, TMACMS, and TMAS:CMS (DM) have similar structures forming aggregates with an overall homogeneous overall appearance. The TMAS-CMS (SD) particles are multi-concave spheres of 1–10 μm with a smooth surface and are well distinct clusters compared to the other derivatives.

### 3.6. Micromeritic Analyses

Considering that the new derivatives are proposed as excipients for monolithic tablets, it was useful to evaluate micromeritic properties (Table 1): Hausner factor (HF), compressibility index (CI), and angle of repose (*θ*), which are directly related to flowability of powders. These parameters are dependent on the size, shape, and rugosity of the grain surface [26]. The angle of repose, also called the angle of friction, was found between 27°–38.2° for all derivatives. TMAS-CMS (SD) had the greatest angle of repose 38.2°. HF was between 1.2–1.4 for all compounds and CI was between 9.5 and 30.5.

### 3.7. Fluid Uptake, Swelling and Erosion

**Figure 5:**
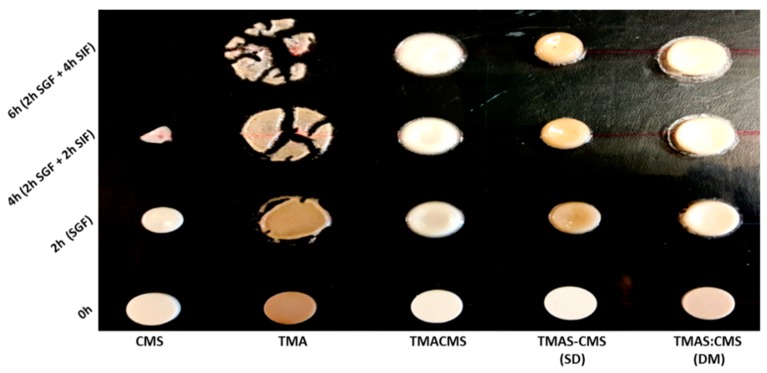
Photographs of placebo tablets of CMS, TMAS, TMACMS, TMAS-CMS (SD), and TMAS:CMS (DM) at 0 h (dried), 2 h in SGF, 4 h (2 h SGF + 2 h SIF), and 6 h (2 h SGF + 4 h SIF).

**Figure 6:**
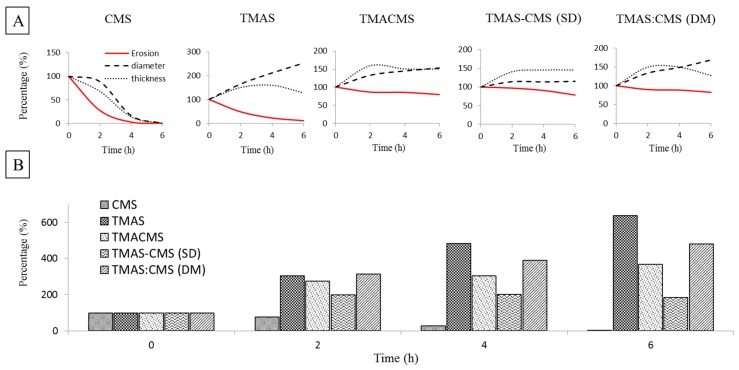
(**A**) Erosion, diameter, and thickness changes of placebo tablets of CMS, TMAS, TMACMS, TMAS-CMS (SD), and TMAS:CMS (DM) at 0 h (dried), 2 h in SGF, 4 h (2h SGF + 2h SIF) and 6 h (2 h SGF + 4 h SIF). (**B**) Fluid uptake of placebo tablets of CMS, TMAS, TMACMS, TMAS-CMS (SD), and TMAS:CMS (DM) at 0 h (dried), 2 h in SGF, 4 h (2 h SGF + 2 h SIF), and 6 h (2 h SGF + 4 h SIF).

Placebo tablets were prepared by direct compression of TMAS, CMS, TMACMS, TMAS-CMS (SD), and TMAS:CMS (DM) powders at 2.5 T/cm^2^. The tablets were immersed in 40 mL of SGF for 2 h and then transferred to 40 mL of SIF for 2 h (total: 2 h SGF + 2 h SIF) and for 4 h (total: 2 h SGF + 4 h SIF). The results are shown in Figure 5. After 2 h in SGF, the CMS tablets were more compact, resulting in a decrease of tablet’s diameter (−11%) and thickness (−27%) without fluid absorption (Figure 6). After transfer to SIF, a rapid erosion was observed with a loss of weight (97.7%) after 4 h and a drastic decrease of size (diameter −86.5% and thickness −93.2%). The tablet was completely disintegrated after 6 h. TMAS displays the highest fluid absorption compared to other derivatives. This phenomenon was pH-independent. After 2 h in SGF the fluid absorption (weight gain) was 305.6% with bidirectional swelling (diameter +64.1% and thickness +50.8%).

After transfer to SIF, the swelling continued, unaffected by pH change, and the tablet had burst after a massive fluid uptake (+482.5% after 4 h and +631.6% after 6 h). The TMACMS had a fluid uptake of 275.4% after 2 h in SGF with an increase of diameter of +23.1% and of thickness +34.6%, swelling and minor erosion (−8.3%). The ampholytic starch TMACMS showed a good stability of weight and shape 6 h after transfer to SIF, with a moderate increase of diameter (+41.3%), thickness (+39.6%), and with a limited erosion (−20.1%) reflecting an independent pH behavior (Figure 6). TMAS-CMS (SD) had the best stability in both SGF and SIF media with diameter had remained stable during the 6 h of exposure. The diameter was 204.4% after 2 h in SFG and showed no significant change after SIF immersion.

Absorption of fluids was proportional to changes in diameter for TMAS-CMS (SD) tablets with +198.2% practically stable for 6 h and an erosion of −21.3% after 6 h. TMAS:CMS (DM) showed a gain of weight 307%, of diameter (+24.9%) and of thickness (+46.4%) after 2 h in SGF. After 6 h in SIF, stability of shapes was observed (Figure 6) with a fluid absorption of 477.6% with diameters (+40.1%) and thickness (+41.6%). TMACMS, TMAS-CMS (SD) and TMAS:CMS (DM) ensured tablet integrity after 6 h (2 h SGF + 4 h SIF). In SGF, a rapid swelling was found but once in SIF, TMACMS, and TMAS-CMS (SD) displayed a better stability than TMAS, CMS, and TMAS:CMS (DM).

### 3.8. In Vitro Dissolution Assays

**Figure 7:**
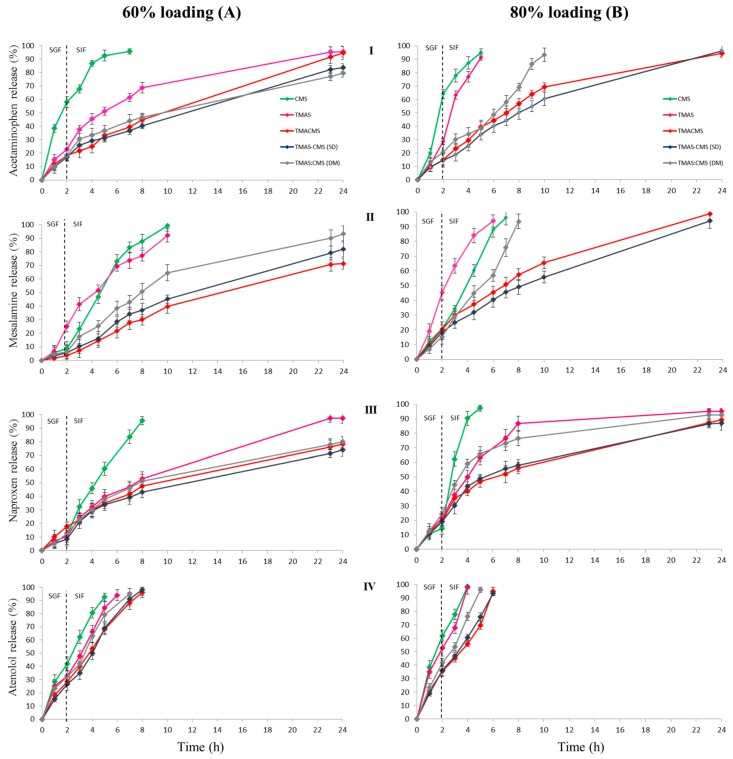
Release profiles of 60% (**A**) and of 80% (**B**) loaded tablets with: acetaminophen (I), mesalamine (II), naproxen (III), and atenolol (IV). The tablets were immersed for 2 h in simulated gastric fluid (SGF) and then transferred to simulated intestinal fluid (SIF).

In order to study the release profiles afforded by the new polyelectrolyte excipients, four APIs (tracers) were selected (one for each BCS class) according to their physicochemical properties. Acetaminophen (class I BCS) has a low sensitivity to the ionic strengths of the medium and pH variations (same solubility in SGF and SIF: 20.3 mg / mL) [26,35]. Mesalamine (class IV BCS) also called 5-Aminosalicylic acid is a monohydroxybenzoic acid carrying an amine in position 5, procuring a zwitterionic nature and an amphoteric behavior with three pKa values: 2.09, 5.26, and 13.64. It is more soluble in SGF (18.2 mg/mL) than in SIF (8.4 mg / mL) [17]. Naproxen (class II BCS) is a chiral molecule derived from propionic acid having a pKa 4.15 [36]. Atenolol (class III BCS) is an isopropylamino-propanol derivative with a pKa 9.6. Monolithic tablets were obtained by direct compression after mixing powders of TMAS, CMS, TMACMS, TMAS-CMS (SD), or TMAS:CMS (DM) polymers, and one of the tracers. High loaded (60% API: 40% polymer) and very high loaded (80% API:20% polymer) tablets were obtained. These tablets were dipped in SGF for 2 h at 37 °C under controlled rotation with sampling for quantification of drug concentration every 1 h. Then the tablets were transferred to SIF at 37 °C. The obtained results are shown in Figure 7.

The dissolution profiles of tablets with 60% acetaminophen loading showed that TMACMS, TMAS-CMS (SD), and TMAS:CMS (DM) prolong API release up to 24h with 91%, 82%, and 80% release respectively (Figure 7). However, TMACMS was the only polymer exhibiting pH-independent behavior and a linear sustained release profile in SGF and in SIF. Differently, the CMS and TMAS were not able to extend the release and to provide linear profiles. For mesalamine (60%), all polymeric excipients, except TMAS, provided a gastro-protection in SGF with less than 10% release of API (Figure 7). Once in SIF, only TMACMS and TMAS-CMS (SD) allowed linear profiles and controlled release of mesalamine up to 71% and 77% respectively after 24 h. Differently, TMAS:CMS (DM) showed a faster release of mesalamine in SIF. For naproxen 60% all the polymers except CMS showed two stages of release: first, a limited release in SGF (8–17%) from 0 h to 2 h followed by a controlled release in SIF (Figure 7). TMAS shows a complete release after 24 h, which was not the case for TMACMS (76.5%), TMAS-CMS (SD) (72%), and TMAS:CMS (DM) (80%). When formulated with different excipients and 60% loading, the atenolol was completely released between 5 h and 8 h.

In order to explore the limits of the novel polymeric excipients and to understand the interactions between polymer–polymer and possibly between API and polymeric excipients, dissolution tests were carried out under the same conditions using highly loaded tablets (80% API and 20% excipient). The release profiles are shown in Figure 7. Despite the very high loading (acetaminophen 80%) TMACMS, TMAS-CMS (SD) displayed similar stable profiles, ensuring controlled release up to 24 h: 92% and 95% respectively (Figure 7). Furthermore, the TMACMS, and TMAS-CMS (SD) tablets with mesalamine 80% showed similar profiles to those of acetaminophen with complete release after approximately 24 h (Figure 7), whereas the CMS, TMAS, and TMAS:CMS (DM) presented complete release at 7 h, 6 h, and 10 h respectively. For naproxen, 80% TMACMS and TMAS-CMS (SD) presented nearly identical profiles in SGF and SIF. Differently, TMAS:CMS (DM) and TMAS allowed a faster release in SIF with 73% and 76.5% of the drug released after 8 h. A complete release of the naproxen was observed with CMS after 5 h (Figure 7). Release profiles of atenolol 80% showed a faster release compared to other APIs. Only TMACMS and TMAS-CMS (SD) were able to prolong the release up to 6 h.

## 4. Discussions

The degree of substitution (DS_coo-_) and (DS_N_^+^_(CH3)3_), pH, and Zeta potential (ζ) were determined. The pH values were close to neutral for all of derivatives. Zeta potential values were in line with the theoretical expectations for CMS due to the presence of a negatively charged CM groups, for TMAS due to the presence of a positively charged TMA groups and for the bifunctional starch TMACMS probably due to the presence of the two types of groups CM and TMA. On the other hand, unexpected negative ζ values observed with TMAS-CMS (SD) and of TMAS:CMS (DM) respectively, could be due to a reorganization of the chains with the orientation of the –COO^−^ polar groups towards the surface contact with the liquid medium [37].

### 4.1. Fourier Transform Infrared (FT-IR)

The FTIR of CMS (Figure 1) is characterized by two specific bands at 1589 and 1415 cm^−1^ attributed to stretching vibration of carboxylic –COO^−^ groups [3,38]. For FTIR spectrum of TMAS, the bands at 1553 cm^−1^ and the shoulder at 1476 cm^−1^ could be ascribed to the vibration of methyl groups carried by nitrogen –N^+^_(CH3)3_ [39,40]. This may confirm the grafting of the cationic (trimethylaminohydroxypropyl) group on the starch backbone [41]. For native starch and all derivatives, bands at 3331 cm^−1^ and 2980 cm^−1^ correspond to –OH and –CH stretching vibrations, whereas bands at 1089 and 1000 cm^−1^ were attributed to –CH_2_–O–CH_2_ stretching vibration [3,31]. For TMACMS starch bands at 1553 cm^−1^ and at 1415 cm^−1^ may confirm a successful synthesis of ampholytic starch. The DS variations would explain the observed difference in bands intensities [42,43].

### 4.2. X-ray Diffraction Analysis

The X-ray diffraction showed that the native starch has a more organized semi-crystalline structure than its derivatives. The presence of double helix (B-type), shown by specific peaks at 17.09°, 22.13°, 23.52°, and 24.08°, corresponds to the crystalline fraction of native starch [9]. Diffractions at 12.9° and at 19.89° correspond to the simple helical (V-type). The spectra of TMAS, CMS, TMACMS derivatives, and of compounds TMAS-CMS (SD) and of TMAS:CMS (DM), showed lesser organization compared to Hylon VII, with an almost total disappearance of crystalline zones (B-type) but with persistence of diffractions at 12.91° and 19.89°, corresponding to V-type structures diffused in more amorphous zones [44]. The loss of crystallinity compared to native starch is probably related to starch disorganization by gelatinization in alkaline medium [9]. Also, the grafting of the functional groups CM and TMA (or both) may induce a reorganization [9,31]. The peaks of TMACMS and of TMAS-CMS SD were broader than those of TMAS and of CMS derivatives carrying only one type of ionic group on polymer chains; the same pattern was observed with TMAS:CMS (DM) preparations. When two ionic groups are simultaneously present and possibly associated, the reorganization of polymer chains would be more difficult due to lesser hydrogen association and more hydration. Consequently, the order degree decreased and the morphologies appeared more amorphous. When CMS and TMAS are co-processed as TMAS-CMS (SD), the rapid elimination of solvent (water) generated a slightly more crystalline structure (the 19.89° peak is higher compared to TMACMS). This is not the case of TMAS:CMS (DM) association with a milder processing and lower hydration.

### 4.3. Thermogravimetric Analyses (TGA)

The first loss of mass observed on all samples (Figure 3) was due to the physical dehydration—water evaporation occurring with the temperature rising up to 120 °C [45,46]; the second stage is chemical dehydration and thermal decomposition. The thermal reactions start at around 300 °C with thermal condensation between hydroxyl groups of starch chains to form ether segments and liberation of water molecules and other small species. Dehydration of neighboring hydroxyl groups in the glucose ring may also occur [47]. The last stage, at a temperature above 500 °C, is attributed to material carbonization [45,46]. The highest TD was exhibited by native starch which has a more stable structure than all compounds. This is well correlated with the XRD data (Figure 2). CMS, TMACMS, TMAS-CMS (SD), and TMAS:CMS (DM) display similar decomposition temperature (Figure 3), suggesting that the presence of hydrophilic carboxyl groups decreases the thermal stability of starch macromolecules [45].

### 4.4. Scanning Electron Microscopy (SEM)

The morphology of native starch is probably related to the structural stability offered by hydrogen bridges and crystal regions (B-type) [26]. The similar morphology of CMS, TMAS, TMACMS, and TMAS:CMS (DM) is due to the disorganization of the native structure of starch caused by gelatinization and by substitution. The presence of charged groups may affect the conformation of the helices and the overall polymeric assembling [26]. The TMAS-CMS (SD) has a specific morphology with aggregated concave (empty) granules probably formed at the end of the spray drying by a rapid water removal [48].

### 4.5. Micromeritic Analyses

For the angle of repose, a value of less than 30° indicates a good flow while an angle greater than 56° indicates a bad flow. The HF values lower than 1.2 indicate a good flow of powders but a poor cohesion and compressibility, whereas a HF greater than 1.2 indicates a poor flow and a good cohesion and compressibility [26,49]. The CI should be equal or less than 20% for powders with optimal flowability [26]. Overall, TMACMS has the best micromeritic properties compared to other derivatives (Table 1).

### 4.6. Fluid Up-Take, Swelling, and Erosion

The three derivatives with ampholytic features TMAS-CMS (DM), TMA-CMS (SD), and TMACMS exhibited different swelling behavior: TMAS:CMS (DM) shows a faster fluid uptake and a greater swelling compared to TMA-CMS (SD) and TMACMS. Furthermore, the thickness of the TMAS:CMS (DM) tablet decreases after transfer from SGF to SIF due to faster hydration. The TMA-CMS (SD) placebo tablets show a stable profile (swelling and erosion) especially in SIF, while TMACMS shows a slight change in size due fluid uptake.

### 4.7. In Vitro Dissolution Assays

The dissolution tests performed with placebo tablets showed that TMACMS, TMAS-CMS (SD), and TMAS:CMS (DM) provided longer tablet integrity, for at least 6 h (2 h SGF + 4 h SIF) compared to CMS and TMAS excipients. In SGF, rapid swelling was found due to protonation of carboxyl groups. Once in SIF, TMACMS and TMAS-CMS (SD) display a better stability than TMAS, CMS, and TMAS:CMS (DM); this could be explained by a stronger electrostatic interaction between cationic and anionic starch derivatives (–COO^−^, –N^+^_(CH3)3_) in an aqueous medium than in the dry phase [50] (Figure 5 and Figure 6). To study the release profiles of APIs, dissolution tests have been carried out using four different tracers; release profiles are shown in Figure 7. The choice of tracers was based on BCS which classifies drugs into 4 categories according to solubility and permeability [51]. For acetaminophen, mesalamine, and naproxen, 60% loading the polyelectrolyte excipients TMACMS, TMAS-CMS (SD), and TMAS:CMS (DM) provided a significantly better release control than the single-charged CMS and TMAS derivatives. This seems to confirm the role of an electrostatic interaction between amine and carboxyl groups, and also, a possible reconfiguration of the polymer structure inducing a more stable structure able to form a more compact matrix and ensure a better-sustained release. In some particular cases, a polymer-API interaction could explain the difference between these release profiles. By its zwitterionic nature, mesalamine has an ionic form in SGF and another one in SIF and this could create mesalamine–polymer electrostatic interactions providing greater stability and less hydration of the dosage form. This would explain why the same polymeric excipients were able to control mesalamine and naproxen release better than that of acetaminophen under the same conditions. Differently, the higher solubility of acetaminophen in SGF and SIF (20.3 mg/mL) may accelerate its release by favoring the tablet hydration. For acetaminophen and mesalamine 80%, release profiles are shown in (Figure 7). The TMAS:CMS (DM) afforded a controlled release for 10 h, suggesting that electrostatic interactions are not the only parameter to ensure the matrix stability. The degree of order/disorder of powders studied by XRD (Figure 2) showed that TMACMS and TMAS-CMS (SD) have more amorphous structures compared to the other compounds. This could be due to the attractions between opposite charges of the carboxylic and amines groups with different localizations on the chains. In addition to the hydrogen bridges, the ionic bonds may provide more stability to the TMACMS and to TMAS-CMS (SD) compounds, which would explain the extended release controlled by these two excipients. The naproxen 80% release patterns (Figure 7) support the hypothesis that the ionic interactions established during the synthesis and drying steps of TMACMS and TMAS-CMS (SD) could modify the starch configuration inducing a more stable structure less susceptible to hydration. For atenolol at 60% and 80% (Figure 7), only TMACMS and TMAS-CMS (SD) were able to afford an extended release up to 8 h and 6 h respectively. Considering the solubility of atenolol as well as the small amount of polymers used (133 mg and 50 mg for 60% and 80% respectively) for 200 mg of API/tablet, these results can be perceived as positive. Moreover, the differences observed between the release profiles with TMACMS and TMAS-CMS (SD) and with the other excipients confirm the hypothesis of electrostatic polymer-polymer interactions and possibly of polymer-drug interactions observed with different tracers.

## 5. Conclusions

Ampholytic TMACMS and co-processed TMAS-CMS (SD) starch polymeric excipients showed the best release profiles, even at very high loading. These excipients would be of interest for sustained release systems, especially for high daily dosages of drugs. Simultaneous presence of opposite charges would provide better polymer stability and slow down tablet hydration. Electrostatic interactions strongly impact the microcrystalline organization, in addition to the hydrogen bridges, enhancing their stability. In some particular cases, polymer–drug interactions would influence release patterns. Further investigations concerning the degree of substitution, the ratio of cationic/anionic charges, and also intra- and inter-molecular interactions, would provide greater clarity in stabilization mechanism of starch compounds.

## Figures and Tables

**Table 1:** Micromeritic properties of starch derivatives. HF: Hausner’s factor; CI: compressibility index.

Polymers	HF	CI (%)	*θ* (°)
CMS	1.3	26.3	27
TMAS	1.4	30.5	31.7
TMACMS	1.2	9.5	29.5
TMAS-CMS (SD)	1.3	21	38.2
TMAS:CMS (DM)	1.3	28	30.5

## Abbreviations

TMAS	trimethylaminestarch
CMS	carboxymethylstarch
SD	spray drying
DM	dry mix
TMACMS	trimethylaminecarboxymethylstarch
BCS	Biopharmaceutical Classification System
API	active pharmaceutical ingredient
DS	degree of substitution
PEC	polyelectrolyte complexes
SMCA	sodium monochloroacetate
GTMAC	glycidyltrimethylammonium chloride
CI	compressibility index
HF	Hausner’s factor
USP	United States Pharmacopeia
SGF	simulated gastric fluid
SIF	simulated intestinal fluid
FTIR	Fourier transform infrared spectroscopy
DRX	X-ray diffraction
TGA	thermogravimetric analysis
SEM	scanning electron microscopy
